# Dynamic DNA Methylation in Plant Growth and Development

**DOI:** 10.3390/ijms19072144

**Published:** 2018-07-23

**Authors:** Arthur Bartels, Qiang Han, Pooja Nair, Liam Stacey, Hannah Gaynier, Matthew Mosley, Qi Qing Huang, Jacob K. Pearson, Tzung-Fu Hsieh, Yong-Qiang Charles An, Wenyan Xiao

**Affiliations:** 1 Department of Biology, Saint Louis University, St. Louis, MO 63103, USA; arthur.bartels@slu.edu (A.B.); qiang.han@slu.edu (Q.H.); pnair2013@gmail.com (P.N.); liam.stacey@slu.edu (L.S.); hannah.gaynier@slu.edu (H.G.); matthew.mosley@slu.edu (M.M.); qiqing.huang@slu.edu (Q.Q.H.); jacob.pearson@slu.edu (J.K.P.); 2 Department of Plant and Microbial Biology, North Carolina State University, Raleigh, NC 27695, USA; thsieh3@ncsu.edu; 3 Plants for Human Health Institute, North Carolina State University, North Carolina Research Campus, Kannapolis, NC 28081, USA; 4 Plant Genetics Research Unit, Donald Danforth Plant Science Center, Midwest Area, Agricultural Research Service, US Department of Agriculture, St. Louis, MO 63132, USA; yong-qiang.an@ars.usda.gov

**Keywords:** DNA methylation, methylome, seed, development, gene expression, dynamics, epigenetics, transposable element, plant

## Abstract

DNA methylation is an epigenetic modification required for transposable element (TE) silencing, genome stability, and genomic imprinting. Although DNA methylation has been intensively studied, the dynamic nature of methylation among different species has just begun to be understood. Here we summarize the recent progress in research on the wide variation of DNA methylation in different plants, organs, tissues, and cells; dynamic changes of methylation are also reported during plant growth and development as well as changes in response to environmental stresses. Overall DNA methylation is quite diverse among species, and it occurs in CG, CHG, and CHH (H = A, C, or T) contexts of genes and TEs in angiosperms. Moderately expressed genes are most likely methylated in gene bodies. Methylation levels decrease significantly just upstream of the transcription start site and around transcription termination sites; its levels in the promoter are inversely correlated with the expression of some genes in plants. Methylation can be altered by different environmental stimuli such as pathogens and abiotic stresses. It is likely that methylation existed in the common eukaryotic ancestor before fungi, plants and animals diverged during evolution. In summary, DNA methylation patterns in angiosperms are complex, dynamic, and an integral part of genome diversity after millions of years of evolution.

## 1. Introduction

DNA methylation generally refers to an addition of a methyl group onto the C5 position of cytosine to form 5-methylcytosine (5mC). DNA methylation is an important epigenetic mechanism that is involved in transposable element (TE) silencing, genome stability, X-chromosome inactivation, and genomic imprinting. DNA methylation in promoters has been shown to regulate gene expression and plays a critical role in the growth and development of plants and mammals. DNA methylation in the symmetric CG context is an evolutionarily conserved modification in plants, mammals and some fungi [1,2,3,4]. In mammals, DNA methylation is initiated by de novo DNA methyltransferase 3 (Dnmt3) [5] and maintained by DNA methyltransferase 1 (Dnmt1) [6]. In higher plants, in addition to CG methylation, DNA methylation also occurs in the CHG (symmetric) and CHH (asymmetric) contexts (H = A, C, or T). In *Arabidopsis*, DNA METHYLTRANSFERASE 1 (MET1), an ortholog of Dnmt1 in mammals, maintains CG methylation [7,8,9,10]. CHROMOMETHYLASE 2 and 3 (CMT2 and CMT3) [11,12,13] and the de novo DNA methyltransferases DOMAINS REARRANGED METHYLTRANSFERASE 1 and 2 (DRM1 and DRM2) [14,15] are mainly responsible for DNA methylation at the CHG and CHH contexts. *Oryza sativa* (rice) has OsMET1-1 and OsMET1-2, *Prunus persica* (peach) has one PsMET, and *Zea mays* (maize) has one ZmMET1 [16,17,18]. For the CMT family, *Brassica rapa* has one BrCMT [19], rice has two OsCMTL and OsMET2a but their functions have not been confirmed, and *Zea mays* has two ZMET2 and ZMET5 [20]. For the DRM family, *Oryza sativa* and *Zea mays* each have two homologous proteins OsDMT106 and OsZmet3, ZmDMT106 and Zmet3, respectively, but their biological functions are not known [21].

DNA methylation used to be thought as static: methylation on DNA remains there after being added by DNA methyltransferases. However, DNA demethylation can occur passively during DNA replication when a newly synthesized strand is not methylated by DNA methyltransferases, or it can occur actively to remove 5-methylcytosines by the base excision repair (BER) pathway in plants. The discoveries of DNA demethylases DEMETER (DME) and REPRESSOR OF SILENCING 1 (ROS1) show that the methylation process can be actively reversed. Both *DME* and *ROS1* encode DNA glycosylase, which catalyzes reactions to actively remove 5-methylcytosine through the BER pathway. Active DNA demethylation has also been found in mammals: demethylation can be achieved by ten-eleven translocation (TET) dioxygenases to form 5-hydroxymethylcytosine (5-hmC) through oxidation of the methyl group, and then 5-hmC is converted into unmodified cytosines by DNA glycosylase-mediated BER. In plants, CHH methylation can occur through the siRNA-mediated RNA-directed DNA methylation (RdDM) pathway. Small, non-coding RNAs (sRNAs, 19–24 nucleotides (nt) in length) play a critical role in growth, development, and stress response in both mammals and plants [22]. The 24-nt sRNAs are produced from double stranded RNA through the activities of RNA-dependent RNA polymerase 2 (RDR2) and DICER-LIKE 3 (DCL3), then bind ARGONAUTE 4 (AGO4) protein [23,24] and recruit DRM2 to catalyze methylation at CHH sites.

Research has shown that DNA methylation regulates leaf morphology, flowering time, floral organ identity, fertility, and embryogenesis in addition to silencing TEs, repetitive sequences, and transgenes in plants [25,26,27,28,29,30,31,32,33,34,35]. Mutations in DNA methyltransferase MET1 and DECREASE IN DNA METHYLATION 1 (DDM1), an ATP-dependent SWI2/SNF2 chromatin-remodeling factor, also affect seed development [33,36,37,38], suggesting that DNA methylation is critical for seed development. *DME* DNA glycosylase is expressed specifically in the central cell of the female gametophyte and vegetative cell of the male gametophyte. DME-mediated DNA demethylation is essential for endosperm development in *Arabidopsis*.

Recent research shows that DNA methylation is dynamic during plant development. For example, DNA methylation levels in the gene promoter can change during different stages of seed maturation in soybean [39]. Furthermore, DNA methylation levels vary in different cell types in gametophytes that have the same origin and are separated only by a few cell divisions. However, the molecular mechanism regulating these dynamic DNA methylation patterns remains to be elucidated.

This review focuses on the variation of DNA methylation in different plant species, organs, tissues, and cells. The dynamics of DNA methylation are also summarized during plant growth and development as well as in response to environmental stresses. Future challenges in this area are also discussed.

## 2. Variations of DNA Methylation in Different Plant Species

**Figure 1:**
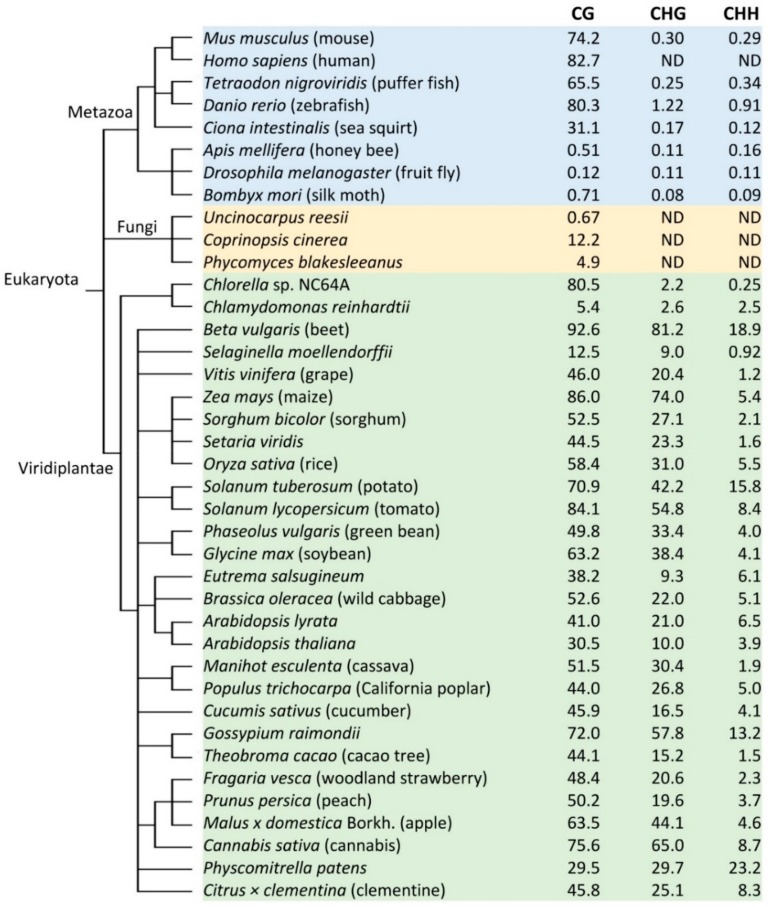
DNA methylation levels in 39 eukaryotic organisms. Although the methylation data were from different studies, and in different organs or tissues that might have some technical variations among different experiments, all of these were from recent methylome research, and procedures and technologies used were similar, including genomic library construction, bisulfite conversion and efficiency, and next-generation sequencing. Taxonomy was obtained from the National Center for Biotechnology Information (NCBI) (https://www.ncbi.nlm.nih.gov/taxonomy). Species, materials and methylation data in Figure 1 were from references listed below: *M. musculus*, E13.5 embryos from strain C57BL/6J [45]; *H. sapiens*, H1 human embryonic stem cells [46]; *T. nigroviridis*, whole fish [44]; *D. rerio*, 5-day-old embryos [45]; *C. intestinalis*, *Ciona* animals collected from Half Moon Bay, CA [45]; *A. mellifera*, whole adult workers [44]; *D. melanogaster*, embryo 0–3 h [44]; *B. mori*, whole larvae [44]; *U. reesii*, mycelium [44]; *C. cinereal*, mycelium of strain Okayama 7 [44]; *P. blakesleeanus*, mycelium of strain NRRL 1555 [44]; *Chlorella* sp. NC64A, cells cultured in medium [44]; *C. reinhardtii*, vegetative cells from strain CC503 [45]; *B. vulgaris*, leaf [41]; *S. moellendorffii*, aerial tissues of adult soil plants [44]; *V. vinifera*, leaf [41]; *Z. mays*, kernel [47]; *S. bicolor*, leaf [41]; *S. viridis*, leaf [41]; *O. sativa*, leaf [41]; *S. tuberosum*, tuber tissue [48]; *S. lycopersicum*, leaf [41]; *P. vulgaris*, leaf [41]; *G. max*, fully expanded leaf [39]; *E. salsugineum*, leaf [41]; *B. oleracea*, leaf [41]; *A. lyrate*, leaf [41]; *A. thaliana*, leaf [41]; *M. esculenta*, leaf [41]; *P. trichocarpa*, leaf [41]; *C. sativus*, leaf [41]; *G. raimondii*, leaf [41]; *T. cacao*, leaf [41]; *F. vesca*, leaf [41]; *P. persica*, leaf [41]; *M. domestica*, leaf [41]; *C. sativa*, leaf [41]; *P. patens*, whole plants growing on plates [44]; *C. clementine*, leaf [41]. ND, not determined.

To date, DNA methylation has been studied in many plant species, ranging from algae, cereal crops, vegetables, to trees. Research shows a wide diversity of DNA methylation in terms of levels and sequence contexts (Figure 1). Among more than 30 plant species with methylome data, non-vascular unicellular *Chlamydomonas reinhardtii* (green algae) has the lowest DNA methylation (5.4%, 2.6%, and 2.5% in the contexts CG, CHG and CHH, respectively) (Figure 1) [40,41]. By contrast, land plants in general have much higher levels of DNA methylation, especially at CG and CHG contexts. For example, in *Arabidopsis* leaves, 30.5%, 10.0%, 3.9% methylation occurs in CG, CHG, CHH sites, respectively [41]. Rice leaves have an intermediate level of DNA methylation: 58.4% of CG, 31.0% of CHG, and 5.1% of CHH sites are methylated [41]. *Beta vulgaris* (beet) leaves have the highest DNA methylation: 92.6%, 81.2%, and 18.9% in CG, CHG and CHH, respectively (Figure 1) [41].

It is apparent that genome-wide DNA methylation shows extensive variation among plant species in all three DNA methylation contexts (Figure 1) [41,42,43]. CG methylation is the predominant type of DNA methylation compared with CHG and CHH methylation. In angiosperms, CG methylation contributes to more than 50% of total cytosine methylation [41]. CHG and CHH methylation levels vary more widely than CG methylation among species. *B. vulgaris* has a very high percentage of CHG (81.2%) and CHH methylation (18.9%), while *Eutrema salsugineum* has the lowest CHG methylation (9.3%) and moderate CG (38.2%) and CHH methylation (6.1%). *Vitis vinifera* has the lowest CHH methylation level (1.2%) with 46.0% CG and 20.4% CHG methylation [41]. Species in Brassicaceae have reduced CG and CHG methylation, while CHH methylation is depleted in some species in Poaceae, which suggests that DNA methylation patterns are diverse in various species [41].

In genic regions, angiosperms and unicellular green algae have a marked dip in DNA methylation just around the transcriptional start site (TSS) and high CG methylation in most gene bodies, while gymnosperms like *Selaginella moellendorffii* lack DNA methylation at the TSS and gene bodies [44,45]. TEs are highly methylated in plants, and methylation is correlated with the inhibition of TEs and repeats. Components in different methylation pathways will be discussed in Section 7.

## 3. DNA Methylation Profiles in Different Plant Organs and Tissues

**Figure 2:**
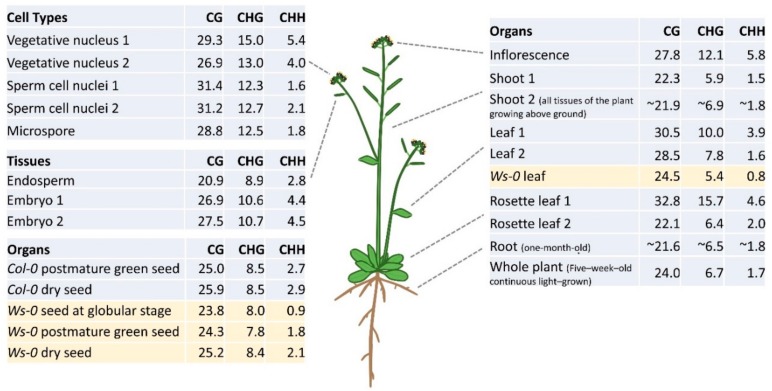
DNA methylation levels in different organs, tissues, and cells in *Arabidopsis*. Organs, tissues, or cell types were collected from wild type *Col-0* (Columbia) except the organs indicated as *Ws-0*, referring to Wassilewskija. Organs, tissues, cells, and methylation data were from references listed below: vegetative nucleus 1, sperm cell nuclei 1, microspore, embryo 2, and inflorescence [61]; vegetative nucleus 2 and sperm cell nuclei 2 [62]; endosperm and embryo 1 [55]; postmature green seed, dry seed, leaf 2, *Ws-0* seed and leaf [63]; leaf 1 [41]; rosette leaf 1 [64]; Rosette leaf 2 [65]; shoot 1 [45]; shoot 2 and root [53]; whole plant [66]. The methylation data were from different studies that might have some technical variations among different experiments, but procedures and technologies (genomic library construction, bisulfite conversion and efficiency, and next-generation sequencing) used were similar, thus overall results can be compared.

Previous studies showed that there is increased DNA methylation in vegetative organs, such as leaves, shoots, and roots compared with cotyledons in many species like tomato, *Silene latifolia*, and *Arabidopsis* [49,50,51]. Juvenile shoot apical meristem (SAM) has lower DNA methylation compared with adult SAM in peach [52]. In *Arabidopsis*, CG methylation levels are very similar among different tissues except for reduced methylation in endosperm although there may be some technical variations among different experiments (Figure 2) [39,53,54,55]. In *Arabidopsis* root meristem, the genome of the columella root cap is the most highly methylated cell characterized [56]. A study shows that CG and CHG methylation levels were very similar among examined organs: embryos, shoots, roots, and leaves in rice [54], but CHH methylation levels increased from embryos to young shoots and roots, and reaches its highest levels in mature leaves [54]. Rice endosperm compared with the embryo has lower DNA methylation in all three methylation contexts, which is the same as *Arabidopsis* (Figure 2) [54,55]. In soybean, the difference of DNA methylation levels in all contexts was small between any two examined vegetative organs [57].

It is not clear if or when global DNA demethylation occurs [58,59]. During development of the male sexual lineage, CG and CHG methylation are kept at high levels in meiocytes, microspores, and sperms compared with those in shoot (Figure 2), which is consistent with a previous study showing high efficiency of CG methylation in pollen (Figure 2) [60]. CHH methylation levels are reduced in the sperm cell nucleus with significant elevation in the microspores and sperms, suggesting a conflict between methylation maintenance and demethylation during the development of the male sexual lineage [58].

The difference in DNA methylation levels of various tissues is likely related to the involvement of different DNA methylation pathways. CG methylation in the loci of some sexual specific lineage is initiated by the RdDM pathway in germ line cells. However, these loci are maintained at a lower CG methylation level in the somatic cells by the MET1 rather than the RdDM pathway [58].

## 4. Reprogramming of DNA Methylation in Gametogenesis

It is controversial whether plants have a segregated germline or when the germline is differentiated during development [67]. Regardless whether a germline in a plant species is set aside early or late during development, the first germline cell likely inherits most of its epigenetic profile from the somatic parent plant. When a megaspore mother cell undergoes meiosis to form four haploid megaspores, one of the four spores develops into a haploid female gametophyte. Recent research clearly indicates that DNA methylation profiles in different cell types of the female gametogenesis undergo reprograming or dynamic changes that results in DNA methylome different from the somatic parental cells [62].

Genomic imprinting refers to differential expression of parental alleles. The central cell is fertilized by a sperm to form the endosperm where most of genomic imprinting occurs. *MEDEA* (*MEA*), *FERTILIZATION INDEPENDENT ENDOSPERM* (*FIE*), *FERTILIZATION INDEPENDENT SEED 2* (*FIS2*), and *FLOWERING WAGENINGEN* (*FWA*) are the first few set of genes that were identified to be maternally expressed imprinted genes [35,68,69,70]. Their methylation status undergoes dynamic changes: methylation of the maternal allele was maintained by MET1 before differentiation of the central cell, and then demethylated by DME in the central cell, resulting in hypomethylation whereas the paternal allele of those genes was methylated by DNA methyltransferases in the male gametophyte. The maternal allele of a gene demethylated by DME in the central cell, and the maternal MET1 allele is repressed by Polycomb Repressive Complex 2 (PRC2) [71,72], thus the maternal allele is hypomethylated and silenced. The maternal *MEA*, *FIE*, *FIS2*, and *FWA* allele expression in the *Arabidopsis* central cell and endosperm is antagonistically regulated by DME and MET1 [26,34,35,69,70]. Some maternal hypomethylated TEs due to *DME* activity can result in expression of small RNA in the endosperm, which is hypothesized to be transported to the embryo to methylate the homologous TEs or nearby genes through the RdDM pathway, reinforcing gene silencing in the embryo [62]. The *maternally expressed in embryo 1* (*mee1*) in maize, is imprinted in both the embryo and endosperm [73]. The embryonic maternal *mee1* is demethylated on fertilization and remethylated during embryogenesis. However, the maternal *mee1* remains hypomethylated in the endosperm [73]. It remains unknown which mechanism causes the maternal demethylation in early embryo. One speculation is whether siRNA generated from the hypomethylated maternal allele in endosperm can mediate recruitment of DME to the maternal *mee1* allele in early embryo.

Epigenetic changes or reprogramming during male gametogenesis result in different epigenetic profiles in gametes compared with a microspore mother cell or its somatic parental cells. In the male gametophyte, *DME* is specifically expressed in the vegetative cell instead of the sperm cell. *DME* expression is limited in late bicellular stage pollen and reduced to undetectable level as pollen matures [74]. Thus, CG methylation in imprinted genes or DME-targets is lost in the vegetative nucleus. Expression of siRNA from hypomethylated TEs in the vegetative nucleus is likely to move from the vegetative nucleus to the sperm cell to reinforce silencing of TEs in the sperm cell which passes its genetic information to the next generation [61,62,75]. CHH methylation in TEs is lost in microspores and sperm nuclei but is restored by 24-nt small RNA in both the vegetative nucleus and fertilized embryo (Figure 2) [61]. Furthermore, epigenetically activated siRNAs from the paternal genome can regulate parental genome dosage and seed viability [76]. Thus, reprogramming of DNA methylation in the male gametophyte (vegetative and sperm cells) and the female gametophyte (the central cell and egg cells) through DNA methyltransferases, demethylases and small RNA is part of the epigenetic mechanisms to maintain overall inheritance of phenotypes to the next generation. It is worth mentioning that not all imprinted genes are a byproduct of epigenetic reprogramming in gametes and seed. Studying imprinting in *Arabidopsis lyrata* shows that the maternal allele of many paternally expressed imprinted gene (PEGs) was hypermethylated in CHG, and this increased CHG hypermethylation was correlated with increased expression bias in favor of the paternal allele, suggesting that CHG methylation in the maternal allele of PEGs reinforces the silencing of the maternal allele [77].

## 5. Dynamic DNA Methylation during Seed Development and Germination

Seed development is vital for seed quality and yield. Research has shown that DNA methylation changes during seed development. During soybean seed development, DNA methylation in the CHH context increased from 6% at the early stage to 11% in the late stage [39]. In soybean, a total of 2136 genes contain differentially methylated regions (DMRs) with a negative correlation between gene expression and CHH methylation levels in promoters [39]. Another study that profiled both soybean and *Arabidopsis* methylomes from the globular stage through dormancy and germination also showed that CHH methylation increases significantly during seed development while no significant changes occurred for methylation in CG and CHG context [63]. To gain further insights on whether CHH methylation does regulate seed development, Lin et al. examined the *Arabidopsis drm1 drm2 cmt2 cmt3* mutant (*ddcc*) that is deficient in methyltransferases required for non-CG methylation [63]. Surprisingly seed development, germination, and seed gene activity seem normal in this mutant, suggesting that CHG and CHH methylation might not play a significant role in seed development or in regulating seed gene activity in *Arabidopsis* even though CHH methylation levels increase as the seed develops. However, the authors did find that more than 100 TEs are transcriptionally de-repressed in *ddcc* seeds, implying that CHG and CHH methylation could simply be a mechanism to reinforce TE silencing [63]. To further investigate the role of DNA methylation for seed development and germination, they looked at the methylation landscapes of 75 genes important for seed development and germination. Unexpectedly, they found that half of the genes are located in genomic regions with undetectable or no methylation known as demethylated valleys (DMVs) [63]. The very low or no methylation status of DMVs did not change from fertilization to germination [63]. Another study agrees with these findings that DNA hypomethylation does not appear to be a major mechanism of gene regulation during germination but could affect the expression of a specific set of genes. The conclusion was based on the fact that most CHH DMRs did not have a complete removal of methylation but a gradual reduction in methylation. This gradual reduction of methylation was viewed as passive DNA demethylation [78].

Hypermethylation could be related to a halt in transcription as dry seeds enter dormancy [79,80]. Examining expression of genes involved in the DNA methylation and demethylation pathways shows that ROS1, DEMETER-LIKE 2 and 3 (DML2 and DML3) are not involved in global demethylation, and demethylation occurs in a passive manner by dilution of methylation because of increased cell division [79]. During *Arabidopsis* seed maturation, nuclear size is reduced and nuclei are highly condensed in seeds while the opposite is observed during germination—nuclei regain their size and chromatin is decondensed [81]. It is likely that DNA methylation is involved in chromatin condensation and decondensation during seed maturation and germination, respectively. In rice seed development, the highest levels of methylation were reached in the endosperm at 2 days after pollination (DAP), when cellularization and genome-wide demethylation began, resulting in increased expression of demethylated genes [82]. This suggests that endosperm cellularization could be regulated by dynamic methylation. Additionally, 25 genes show differential methylation during rice seed development [82]. More significantly, recent research in *Brassica rapa* clearly shows that DNA methylation is required for seed development [83]. Mutations in Pol IV-mediated small RNA pathway result in defects in reproduction of *Brassica rapa*. Furthermore, The RdDM pathway is crucial in maternal somatic tissues, not in the female gametophyte or zygote [83]. This suggests that different plant species might have different sensitivity or tolerance to loss or interruption of DNA methylation.

## 6. Alteration of DNA Methylation in Response to Environmental Stimuli

DNA methylation is involved in plant response to environmental stresses. By whole-genome bisulfite sequencing of single-cell root hairs and multicellular stripped roots in response to heat stress in soybean, it was found that both samples showed hypomethylation after stress. Although the differences between the control and stressed samples were marginal (less than 10%) among CG and CHG, CHH methylation in stressed samples decreased significantly by 25% and 37% in genes and TEs, respectively [84]. When compared to wild-type plants, the mutant of *ddm1* plants had shorter roots and lower survivability during salt stress [85]. This could be due to the alteration of expression of transcription factors since they have been shown to play an important role in gene activation during salt stress [86]. *AtMYB74*, a transcription factor of the *R2R3-MYB* gene family, had a significant increase in the mRNA level in response to salt treatment, and levels of DNA methylation were significantly reduced in the *AtMYB74* promoter. Expression of *AtMYB74* and its promoter methylation is regulated by the 24-nt siRNAs-mediated RdDM pathway during salt stress [86]. Evidence that epigenetics plays a role in salt tolerance extends to the *Arabidopsis* H3K4 demethylase gene *JMJ15*. Gain-of-function mutants showed enhanced salt tolerance and loss-of-function mutants were more sensitive to salt [87]. Plants under a mild drought stress accumulated drought-associated epialleles, but the epialleles were not correlated with gene expression of drought-responsive genes [65]. Under transgenerational drought stress, negligible conserved differentially methylated regions (DMRs) were observed in drought-exposed lineages compared with control plants, suggesting that DNA methylome is relatively stable under drought stress [65].

DNA methylation has been linked to plant immune response [88,89]. Studying DNA methylation in response to different virulent factors showed that mutants with reduction of cytosine methylation (*met1* and *drm1 drm2 cmt3* (*ddc*)) were more resistant to *Pseudomonas syringae* pv *tomato* DC3000 (*Pst*DC3000) infection [90]. Expression of many pathogen-responsive genes was altered in the mutants. These data suggest that certain genes were repressed by DNA methylation in non-affected tissues, but upon infection by pathogens, methylation could be changed to adapt to stress condition. Methylation in the CG and CHG contexts was altered similarly but methylation in the CHH context varied when exposed to different pathogens, suggesting that different pathogens can cause distinct changes of CHH methylation levels.

Although methylation profiles can change in response to biotic stresses such as pathogen attack, whether alteration of DNA methylation can allow plants to prime their descendants for disease-resistance transcriptional changes needs to be substantiated [91]. By comparing the progeny of *Arabidopsis* treated with *Pst*DC3000 and control plants, the *Pst*DC3000-treated *Arabidopsis* had progeny that were primed to activate the salicylic acid-inducible defense genes and were more resistant to the pathogen *Hyaloperonoospora arabidopsis* (*Hpa*) and *Pst*DC3000 [92]. The *ddc* mutant had progeny that mimicked the resistance, suggesting that DNA hypomethylation and non-CG methylation might serve as the transmitter of immunity to the next generation in plants. Recently it has been shown that DNA methylation and DNA demethylation can have opposite effects on basal resistance to *Hpa* [93]. Several hypo-methylated mutants including *nrpe1* (*nuclear RNA polymerase E1*) displayed enhanced resistance to *Hpa* while two hyper-methylated mutants including *ros1* were more susceptible to the pathogen. It is exciting to see this emerging evidence that priming can alter plant epigenetic profiles and potentially improve plant resistance to stresses especially biotic stress. However, occurrence and inheritance of such an epiallele that allows plants transgenerational priming are usually a very rare sporadic event [91,94,95]. Mechanistically an epiallele can be generated through the movement of TEs and altered DNA methylation patterns via the RdDM pathway, or an epigenetic byproduct of an aggressive germline defense strategy. To utilize these epialleles to benefit agriculture in genetics and breeding will remain challenging because it is very unlikely these induced epigenetic alterations in response to environmental stresses are transgenerationally inherited.

## 7. Mechanisms of Dynamic DNA Methylation

Cytosine methylation is an ancient modification that is required to maintain genomic structure and stability in many eukaryotes. In plants, DNA methylation has been found to silence TEs and repeats as well as regulate gene expression. Animals have a separate germ line where DNA methylation patterns are erased and reestablished. It has been debatable whether plants have a segregated germline established early in development [67], but most plants are thought to set aside a germline cell (megaspore or microspore mother cells) late in development and there is no genome-wide erasure and reestablishment during gametogenesis in plants, meaning that epigenetic changes induced in parents can be inherited and maintained in progenies [96]. This might allow plants to find a balance between keeping epigenetic patterns stable to avoid detrimental effects on genome structures and keeping those epigenetic patterns sufficiently flexible to induce epigenetic variation required for quick adaptation to new environmental conditions [97]. These dynamic methylation patterns depend on the coordination of many plant-specific methyltransferases and demethylases.

*MEA*, a SET domain Polycomb group gene, was the first plant gene to be identified as imprinted in the endosperm. MET1 is responsible for maintaining methylation at the *MEA* promoter, and DME DNA glycosylase antagonistically excises 5-methylcytosine at the maternal *MEA* allele through the BER pathway [34]. Mutations in *ROS1* cause transcriptional gene silencing of transgenes [98]. Active DNA demethylation by DME and ROS1 prevents accumulation of 5-methylcytosines at genes. Interestingly, *ROS1* expression is induced by DNA methylation and suppressed by DNA demethylation [99]. Induced methylation in the *ROS1* proximal region can restore *ROS1* expression in an RdDM mutant. It was suggested that *ROS1* functions as an epigenetic rheostat to maintain epigenome stability by adjusting ROS1 demethylase activity in response to methylation changes [99,100].

The RdDM pathway is a major mechanism for cytosine methylation in euchromatic TEs in *Arabidopsis* and maize. RdDM is initiated by the transcription of non-coding single-stranded RNAs (ssRNAs) by a plant-specific RNA polymerase, RNA Pol IV. Then, these non-coding ssRNAs are used to synthesize double-stranded RNAs (dsRNAs) by RNA-dependent RNA polymerase 2 (RDR2). Afterwards, DICER-LIKE 3 (DCL3) will process the dsRNA [54] into 24-nucleotide siRNAs, which are then methylated at their 3′-OH by HUA ENCHANCER 1 (HEN1) and loaded onto ARGONAUTE 4 (AGO4) [101]. Pol V recruits AGO4 through the C-terminal domain of Pol V’s largest subunit, NRPE1, which is able to interact with the KOW DOMAIN-CONTAINING TRANSCRIPTION FACTOR 1 (KTF1), a transcription factor that contains an AGO hook motif [102,103,104]. Recently it has been shown that CLASSY (CLSY) 1–4, SNF2-related, putative chromatin remodeling factors, are required for locus-specific and global DNA methylation in *Arabidopsis* [105]. The CLSY family controls 24-nt-siRNA production and is crucial for Pol IV chromatin association [105]. Finally, the AGO4-bound siRNA is proposed to pair complementarily with the Pol V transcript and recruit DRM2 to catalyze de novo methylation at homologous genomic sites.

The RdDM pathway has been shown to transcriptionally repress TEs and alter gene expression that is partially involved in pathogen defense, stress responses, development, and intercellular communication. While there has been no obvious fertility defect associated with loss of RdDM in *Arabidopsis*, the maize *ago9* mutant failed to complete meiosis and generate functional gametes [106]. The RdDM pathway regulates parental gene imprinting at several loci in *Arabidopsis* [107]. Furthermore, the maternal allele of components in the RdDM pathway is required for seed development in *Brassica rapa* [83]. The RdDM pathway has also been shown to respond to environmental changes, which in turn triggers epigenetic changes at particular loci to generate heritable epialleles in the next generation [108,109]. In short, the RdDM pathway might represent a form of epigenetic adaptive inheritance that could offer fitness advantage to descendants after a plant encounters a particularly stressful environment [92,110].

## 8. Conclusions and Discussion

Since the first eukaryotic DNA methylome was sequenced in base-pair resolution in *Arabidopsis* 10 years ago [66,111], DNA methylomes of more than 50 organisms have been sequenced including many plant species, which greatly increases our knowledge about DNA methylomes. In angiosperms, genome-wide DNA methylation levels are thought to be correlated with genome size for CG and CHG, but not for CHH. However, after removing DNA methylome data of *Z. Mays* (the largest genome with the whole methylome data), only genome-wide CHG methylation levels remain correlated with genome size [41], while CG and CHH methylation show no correlation with genome size. This seems counterintuitive since CG are highly methylated in repetitive sequences that usually increase as genome size increases. Alternatively, it can be due to difference of GC contents in a genome, or particular regions in the genome, and/or chromatin structure such as histone variant H2A.Z distribution [44,112]. Studies have revealed a wide variation of DNA methylation among different species, organs, tissues, and cells. Existing hypotheses such as correlation between genome size and genome-wide DNA methylation levels, GC contents, and chromatin structure cannot fully explain why there is such a big variation of DNA methylation among different species. For example, *C. elegans* has no DNA methylation, *Drosophila melanogaster* has very low or no detectable methylation, some fungi have relatively low methylation, and angiosperms and mammals have relatively high methylation. There are also no consistent patterns in terms of DNA methylation in genes, TEs, and repeats among different organisms. Having said the above, DNA methylation seems to be an ancient part of speciation during eukaryotic evolution or at least an integral part of historical, natural variations in forming different species [40,41,42,44,113].

Although overall DNA methylation is quite diverse among species, it occurs in the CG, CHG, CHH contexts of gene bodies, TEs and repeats in most plants. The Brassicaceae have reduced CHG methylation and reduced or no CG body methylation [41]. The Poaceae have reduced or no CHH methylation in heterochromatin but increased CHH methylation in genic regions [41]. In general, moderately highly expressed genes (e.g., 70th transcription percentile in *Arabidopsis* and rice) are most methylated in the gene body [44]. DNA methylation dips significantly just upstream of the transcriptional start site (TSS) and around the transcriptional termination site (TTS). Low methylation in the promoter is correlated with increased expression for some genes in *Arabidopsis*, soybean, and rice [39]. Three basal plant species *Selaginella moellendorffii*, *Physcomitrella patterns*, and *Marchantia polymorpha* have little methylation or virtually no methylation in gene bodies and around the TSS, but methylation is found in TEs and repeats [44]. This suggests DNA methylation is not part of gene regulation although it may still function to silence TEs and repeats in these diverging land plants. The green alga *Chamydomonas* has an unusual DNA methylation pattern: having relatively high CHG and CHH methylation in gene exons rather than in TEs and repeats [40]. In the angiosperms, DNA methylation functions as an epigenetic mechanism in regulating gene expression in addition to silencing TEs and repeats.

The function of high methylation levels in TEs is to silence the TEs in most plant species. CG methylation is extremely high (mostly higher than 80%) in repeats across all angiosperms, while CHG and CHH methylation varies among species [39,40,41,44,63]. CHG methylation is relatively high, and varies among most angiosperms from 15.2% to 81.2% with the exception of the lowest (9.3%) in Brassicaceae. The amount of CHH methylation is low, and the lowest levels (1.2%) are found in most Poaceae [41]. Genome-wide methylation levels in the CG and CHG contexts are correlated with the proliferation of repeat sequences while CHH methylation is not [41]. It seems that methylation in TEs and repeats is conserved among plants. When horizontal gene transfer occurs among species, a gene duplication event or a whole genome duplication event (e.g., from diploid *Arabidopsis thaliana* to tetraploid *Arabidopsis lyrata*), DNA methylation is involved to methylate and silence the newly duplicated gene or fragments [114]. Occasionally, hypomethylation in the duplicated genes can also occur. Thus, it is likely that DNA methylation divergence in different angiosperms affects gene expression and eventually becomes part of epigenome diversity during evolution.

During eukaryotic evolution it seems that DNA methylation existed very early, perhaps in the common ancestor before fungi, plants, and animals diverged [44]. DNA methylation then follows a complex pathway to evolve in different species. The main driving force of DNA methylation is to limit proliferation following expansion and contraction of TEs. During the process of serving as TE surveillance, it occasionally evolves as an epigenetic gene regulation. For example, maize is a species that has the largest genome among sequenced methylome. Despite its large genome size, the majority of genes are associated with TEs, which are natural targets of DNA methylation. This explains why there are such high levels of methylation in maize (86%, 74%, and 5% in CG, CHG, and CHH, respectively). It is possible that these methylation sites in TEs eventually became an integral part of gene regulation in some specific tissues or cells after millions of years of evolution. DNA methylation can be altered by different environmental stimuli, such as pathogens and abiotic stresses. These dynamic temporary methylation changes might not be preserved during evolution unless plants encounter a constant selection pressure [113]. Different plant species do not have the same or similar DNA methylation patterns because they possess different sets of DNA methyltransferases (MET1, CMT2, CMT3, DRM1, and DRM2) and demethylases (*DME*, *ROS1*, DML2 and DML3). Of course, the RdDM pathway provides another mechanism to methylate and then silence TEs, repeats, and genes. In conclusion, DNA methylation patterns in angiosperms are complex, dynamic, and an integral part of the epigenome during evolution.

## Abbreviations

5mC	5-methylcytosine
AGO	ARGONAUTE
CLSY1-4	CLASSY1-4
CMT2	CHROMOMETHYLASE 2
CMT3	CHROMOMETHYLASE 3
DCL	DICER-LIKE
*ddc*	the *drm1 drm2 cmt3* mutant
*ddcc*	the *drm1 drm2 cmt2 cmt3* mutant
DME	DEMETER
DML2	DEMETER-LIKE 2
DML3	DEMETER-LIKE 3
Dnmt1	DNA methyltransferase 1
DRM1	DOMAINS REARRANGED METHYLTRANSFERASE 1
DRM2	DOMAINS REARRANGED METHYLTRANSFERASE 2
KTF1	KOW DOMAIN-CONTAINING TRANSCRIPTION FACTOR 1
MEG	maternally expressed imprinted gene
MET1	DNA METHYLTRANSFERASE 1 in plants
NRPE1	NUCLEAR RNA POLYMERASE E1
PcG	Polycomb group proteins
PEG	paternally expressed imprinted gene
RdDM	RNA-directed DNA methylation
RDR	RNA-dependent RNA polymerase
ROS1	REPRESSOR OF SILENCING 1
siRNA	small interference RNA
TET	Ten-eleven-translocation enzyme
TSS	transcriptional start site
TTS	transcriptional termination site
